# Long Longitudinal Tract Lesion Contributes to the Progression of Alzheimer's Disease

**DOI:** 10.3389/fneur.2020.503235

**Published:** 2020-10-16

**Authors:** Caimei Luo, Mengchun Li, Ruomeng Qin, Haifeng Chen, Lili Huang, Dan Yang, Qing Ye, Renyuan Liu, Yun Xu, Hui Zhao, Feng Bai

**Affiliations:** ^1^The State Key Laboratory of Pharmaceutical Biotechnology, Department of Neurology, Affiliated Drum Tower Hospital of Medical School, Institute of Brain Science, Nanjing University, Nanjing, China; ^2^Jiangsu Key Laboratory for Molecular Medicine, Medical School of Nanjing University, Nanjing, China; ^3^Jiangsu Province Stroke Center for Diagnosis and Therapy, Nanjing, China; ^4^Nanjing Neuropsychiatry Clinic Medical Center, Nanjing, China

**Keywords:** Alzheimer's disease, white matter damage, cognitive impairment, diffusion tensor imaging, support vector machine

## Abstract

**Background:** The degenerative pattern of white matter (WM) microstructures during Alzheimer's disease (AD) and its relationship with cognitive function have not yet been clarified. The present research aimed to explore the alterations of the WM microstructure and its impact on amnestic mild cognitive (aMCI) and AD patients. Mechanical learning methods were used to explore the validity of WM microstructure lesions on the classification in AD spectrum disease.

**Methods:** Neuropsychological data and diffusion tensor imaging (DTI) images were collected from 28 AD subjects, 31 aMCI subjects, and 27 normal controls (NC). Tract-based spatial statistics (TBSS) were used to extract diffusion parameters in WM tracts. We performed ANOVA analysis to compare diffusion parameters and clinical features among the three groups. Partial correlation analysis was used to explore the relationship between diffusion metrics and cognitive functions controlling for age, gender, and years of education. Additionally, we performed the support vector machine (SVM) classification to determine the discriminative ability of DTI metrics in the differentiation of aMCI and AD patients from controls.

**Results:** As compared to controls or aMCI patients, AD patients displayed widespread WM lesions, including in the inferior longitudinal fasciculus, inferior fronto-occipital fasciculi, and superior longitudinal fasciculus. Significant correlations between fractional anisotropy (FA), mean diffusivity (MD), and radial diffusion (RD) of the long longitudinal tract and memory deficits were found in aMCI and AD groups, respectively. Furthermore, through SVM classification, we found DTI indicators generated by FA and MD parameters can effectively distinguish AD patients from the control group with accuracy rates of up to 89 and 85%, respectively.

**Conclusion:** The WM microstructure is extensively disrupted in AD patients, and the WM integrity of the long longitudinal tract is closely related to memory, which would hold potential value for monitoring the progression of AD. The method of classification based on SVM and WM damage features may be objectively helpful to the classification of AD diseases.

## Introduction

Alzheimer's disease (AD) is a chronic neurodegenerative disease characterized by progressive cognitive decline in multiple domains, including memory, language, executive function, and attention. AD accounts for 60–70% of the ~47.5 million dementia cases worldwide (1), exerting huge distress to patients and their families, as well as an extraordinary financial burden. Amnesic mild cognitive impairment (aMCI) is a prodromal stage of dementia, between normal aging and very early dementia, of which 10–15% progress to AD annually (2). Since there is no cure for AD, there is a great need for monitoring markers in the earlier phase of the disease.

The etiology and pathogenesis of AD are still unsolved. Gray matter neurodegeneration theory has attracted the most attention, particularly the decrease of hippocampal volume (3). Recently, however, there has been an increasing interest in the potential contributions of white matter (WM) integrity damage to the pathogenesis of AD (4, 5). Evidence from the autosomal-dominant AD revealed that WM hyperintensities were a core feature in the process of AD, which can impair cognition either directly, or indirectly by interacting with tau pathology (6, 7). Moreover, the tract-specific WM hyperintensities volume rate was negatively associated with the functional connection in the corresponding connected brain regions (8). The gradual loss of morphological and functional integrity of cortico-cortical pathways [e.g., corpus callosum (CC), inferior fronto-occipital fasciculi (IFOF), superior longitudinal fasciculi (SLF), and inferior longitudinal fasciculi (IFL)] and limbic pathways (e.g., fornix and cingulum) were dominant features accompanied with AD pathogenesis (9). To our knowledge, few studies have investigated the role of cortico-cortical WM integrity on memory processes in patients with aMCI or AD.

Diffusion tensor imaging (DTI) provides an indirect method of detecting a neuroanatomical structure on a microscopic level using water molecules' degree of anisotropy and structural orientation within a voxel (10), which is sensitive to the alterations in the WM structure such as myelin loss, axonal injury, cell death, and edema (11). The parameter of fractional anisotropy (FA) reflects the integrity of the axon. Decreased FA values indicate axonal damage. Mean diffusivity (MD) indicates the rate of molecular diffusion, which increases along with the WM injury (10, 12, 13). Axial diffusion (AxD) reflects the diffusion of water molecules parallel to the axon direction. The radial diffusion (RD) reflects the diffusion of water molecules perpendicular to the axon direction.

Region of interest (ROI) based analysis, voxel-based analysis (VBA), and tract-based spatial statistics (TBSS) are all methods widely used in DTI studies (10). ROI-based methods are highly subjective and only focuses on a certain fiber bundle or a few fiber bundles. Due to requiring a prior assumption, describing the intrinsic WM lesions with ROI-based methods are difficult and may result in poor repeatability. Meanwhile, the VBA method also exhibits some problems, such as registration irregularity and smooth kernel selection (14). Superior to ROI and VBA, TBSS analyzes WM lesions using standard registration algorithms, which do not require prior assumptions, smoothness, or data distribution (14, 15).

The support vector machine (SVM) approach represents a data-driven method for solving classification tasks (16). This approach for neuroimaging data has been widely applied to diagnose individual-level patients. Compared to classifiers based on other methods like decision trees and artificial neural networks, SVM has certain advantages such as high accuracy, avoids less overfitting, and direct geometric interpretation (16). Several studies have used the SVM to classify AD and healthy control based on the features screened from structural MRI (17, 18). The results have shown a high performance with accuracy of up to 92.48%. Until now, few studies have focused on the classification effect based on information from DTI data in the AD spectrum. Therefore, our study aimed to explore the pattern of WM microstructure changes in AD-spectrum patients using the TBSS method. Moreover, SVM was performed to determine the discriminative ability of these DTI metrics in separating aMCI and AD from controls.

## Materials and Methods

### Participants

Overall, 78 right-handed subjects were recruited from the Neurological Department of Nanjing Drum Tower Hospital from September 2016 to December 2018. Among them, 20 subjects were AD patients, 31 subjects were aMCI patients, and 27 subjects were healthy elderly controls. The research protocol received approval from the ethics committees of the Affiliated Drum Tower Hospital of Nanjing University Medical School (clinical trials government identifier: NCT01364246), and each participant provided written informed consent before the experiment. A series of standardized clinical evaluations were arranged, including an interview to obtain medical records, a detailed neuropsychological test battery, a whole-brain 3.0T MRI scan, a general medical examination, and an integrated neurological examination which was implemented by an experienced neurologist (Dr. Zhao).

The clinical diagnosis of aMCI was based on the recommendations of previous studies (19–21), which were as follows: (1) chief complaint of memory impairment, corroborated by the subject and/or an informant; (2) objective impaired memory function documented by an auditory verbal learning test—Huashan 20-min delayed recall (AVLT-DR) score ≤ 1.5 *SD* of age and education adjusted norms; clinical dementia rating scale—sum of the boxes (CDR-SB) score = 0.5 (with a score of at least 0.5 on the memory domain); (3) normal general cognitive functions evaluated by a mini-mental state examination (MMSE) score ≥24; (4) preserved basic activities of daily living or minimal impairment in complex instrumental functions; (5) not diagnosed with dementia. Patients with AD met the National Institute of Neurological and Communicative Disorders and Stroke and the Alzheimer's Disease and Related Disorders Association (NINCDS-ADDRA) criteria for probable AD (22). The criteria for NC participants were as follows: (1) no concerns of cognitive impairment; (2) normal in the neurological examination and cerebral MRI scan; (3) MMSE scores ≥28, Montreal cognitive assessment (MoCA) scores ≥25, CDR scores = 0, and other scores of neuropsychological battery (as described in Part 2.2) within the normal range.

The exclusion criteria applied to all subjects were as follows: (1) vascular cognitive impairment (Hachinski Ischemic Scale score > 4 points), or other types of dementia; (2) depression or other mental disorders, or a history of drug or alcohol addiction; (3) other central nervous system diseases that impact cognitive decline (e.g., epilepsy, Parkinson's disease, or encephalitis) or systemic diseases that interfere with cognitive function (e.g., thyroid dysfunctions, vitamin b12 or folacin deficiency); (4) severe end-stage disease or severe diseases in acute stages; (5) visible WM hyperintensities higher than Fazekas I grade (23); (6) any contraindication for MRI or poor images quality; (7) <6 years of education.

### Neuropsychological Evaluation

A standardized neuropsychological test battery was performed by a professional neuropsychologist who was blinded to the MR imaging results. The Chinese version of the MMSE, the Beijing version of MoCA (24), and CDR (25) scores were used for general cognitive screening. Depression was assessed by the Hamilton depression rating scale (HAMD). Daily life ability was assessed by the activities of daily living scale (ADL). Memory was assessed using the AVLT-DR test and auditory verbal learning test recognition (AVLT-R) (20). The animal fluency test (AFT) and Boston naming test (BNT) were used for language domain assessment. Executive function was evaluated using the trail making test B (TMT-B) and the Stroop-C test. Processing speed was assessed using the trail making test A (TMT-A) and the Stroop-B test. The raw scores of AFT, BNT, TMT-B, Stroop-C, TMT-A, and Stroop-B were transformed to a Z standardization value by the following rule: standardized language (Z-lang) = (Z-AFT+Z-BNT)/2; standardized executive function (Z-EF) = (Z-TMT-B+Z-Stroop-C)/2; standardized processing speed scores (Z-proc) = (Z-TMT-A+Z-Stroop-B)/2. Due to severe dementia, some patients in the AD group failed to complete the TMT test within 10 min, which resulted in missing data.

### MRI Data Acquisition

Whole-brain MRI scanning was performed on a 3.0 T scanner (Achieva 3.0 T TX, Philips Medical Systems, the Netherlands, equipped with an 8-channel head coil). During scanning, cushions and headphones were used to immobilize the subject's head and reduce scanner noise. The conventional three-dimensional T1-weighted acquisition was performed for anatomical reference, using magnetization-prepared rapid gradient-echo sequence with the following parameters: repetition time (TR)/echo time (TE) = 9.8/4.6 Ms, flip angle (FA) = 8°, in-plane resolution = 1.0 mm^2^, field of view (FOV) = 256 × 256 mm, matrix = 256 × 256, and 192 sagittal slices, slice thickness = 1 mm. Diffusion-weighted images were acquired using a spin-echo planar imaging (EPI) sequence (TR/TE = 9,154 /5 ms, FOV = 224 × 224 mm, matrix size 112 × 112, voxel size 2 × 2 × 2 mm^3^, slice thickness = 2.5 mm) with both 32-directional diffusion encoding (b = 1,000 s/mm^2^ for each direction) and no diffusion encoding (b = 0 s/mm^2^). In addition, fluid-attenuated inversion recovery (FLAIR) sequence images were collected to exclude organic physical illness or WM hyperintensity lesions and were obtained with the following parameters: TR/TE = 4,500/344 ms, FA = 90°, matrix = 272 × 272, slice thickness = 1.0 mm, slice number = 200.

### Image Preprocessing and TBSS

Before any pre-process procedures, visual inspection was conducted for all images' artifact, signal-noise ratio, and head motion for quality control. The imaging data were preprocessed using the FSL toolbox (http://fsl.fmrib.ox.ac.uk/fsl/fslwiki) (26, 27). Preprocessing steps comprised of the following: (1) NIFTI format images were converted from the DICOM format; (2) corrected for head motion and eddy current distortions using affine registration in Eddy Current Correction; (3) non-brain voxels were extracted using the Brain Extraction Tool (28) (included in the FSL package) with an extraction factor of 0.2 to generate a binary brain mask; (4) voxel-wise diffusion parameters, including FA, MD, RD, and AxD were then calculated using DTIFIT. All individual FA, MD, RD, and AxD images were performed using TBSS in the FMRIB software library (http://fsl.fmrib.ox.ac.uk/fsl/fslwiki/TBSS/UserGuider). Detailed methods were introduced in the article by Smith et al. (14). The main steps were as follows: (1) each individual original FA map was aligned to a target FMRIB58_ FA template in the MNI space using fully non-linear registrations; (2) all co-registered FA maps were averaged to produce a mean FA image and a mean FA skeleton which represents the centers of all tracts common to the group (using a threshold of mean FA at 0.2 to include non-skeleton voxels); (3) individually aligned FA images were projected onto the mean FA skeleton; (4) the resulting skeletonized FA images were used for voxel-wise cross-subject statistical analysis. Diffusivity maps for MD, RD, and AxD were generated by applying the same steps outlined above.

### Support Vector Machine-Based Classification

SVM has been extensively applied in disease classification. Generally, the SVM procedure involves three stages: feature selection, classifier training, and predication. SVM starts with the feature selection as the basis for classification to form a high dimensional space. Then SVM conducts the classifier training to construct a hyperplane that optimally separates the classes. Lastly, the classifier is used to predict the class label when a new sample is added to the classifier. The validation testing and training dataset is completely independent. In this study, the SVM analysis was performed based on the combined mean FA and mean MD values of clusters which showed significant group differences using the LIBSVM toolkit (https://www.csie.ntu.edu.tw/~cjlin/libsvm/). Due to the small sample size, we used the leave one out cross-validation (LOOCV) test to evaluate the average discriminant accuracy among the three groups. This strategy provides an optimistic estimation of accuracy since only one subject is left out for testing while the others are used to train the classifier and get the optimal parameters, just as mentioned in Weikai et al.'s research (29, 30). The performance of a classifier can also be quantified using sensitivity, specificity, and the area under the receiver operating characteristic curve (ROC) according to the results of cross-validation.

### Statistical Analysis

Voxel-wise statistical analysis of FA, MD, RD, and AxD maps was conducted using randomize (part of FSL toolbox), a non-parametric permutation program inference on statistic maps when the null distribution is ambiguous. A standard general linear model (GLM) design matrix was applied across all subjects to identify the significant regions among three groups with age, gender, and years of education controlled as possible confounding variables. Random permutations were 5000, *P* < 0.01, using threshold-free cluster enhancement (TFCE) for multiple comparison corrections. Mean FA, MD, RD, and AxD values of significant clusters on each tract (anatomical locations of the significant clusters were defined by ICBM-DTI-81 white-matter labels atlas and JHU white-matter tractography atlas) were extracted based on the fslstats command. The following WM tracts were discussed in our present research: anterior thalamic radiation (ATR); corticospinal tract (CST); cingulum of the cingulate gyrus (Ccing); cingulum of the hippocampus (Chippo); forceps major (for ma); forceps minor (for mi); IFOF; IFL; SLF; uncinate fasciculus (UF); superior longitudinal fasciculus-temporal part (SLFt); and CC. Mean FA, MD, RD, and AxD values of each significant cluster were analyzed using ANOVA and *post-hoc* analyses (using the Bonferroni correction).

Statistical analysis of demographic, clinical variables, and neuropsychological scores was all performed using SPSS version 20 (SPSS, Chicago, IL, U.S.A). Continuous variables were tested by one-way ANOVA (LSD for *post-hoc* analysis) or a Kruskal–Wallis test (non-normally distributed variables). Categorical variables were compared using the Chi-squared test or Fisher's exact test. Partial correlation analysis was performed to detect the relationship between diffusion metrics of a cluster which showed significant group differences and the neuropsychological scores in aMCI and AD groups, respectively, controlling for the effect of age, gender, and years of education. Power analysis was performed in the G power 3.1.9.2 version. *P* < 0.05 was statistically significant.

## Results

### Demographics and Clinical Characterizations

No significant differences were found in gender, age, HAMD, hyperlipidemia ratio, hypertension ratio, or diabetes ratio among the three groups (Table 1). However, the individuals in the AD group had significantly lower years of education than those in the NC and aMCI groups (*P* = 0.046, Table 1). Significant differences in the MMSE, MoCA, ADL, CDR, AVLT-DR, AVLT-R, and Z-lang scores were found among the three groups (for details see Table 1). There was no difference in the scores of executive function and processing speed between the NC and aMCI groups.

**Table 1:** Demographic and clinical characteristics of all subjects.

	**NC (*n* = 27)**	**aMCI (*n* = 31)**	**AD (*n* = 20)**	***P***	***Power***
Age (years)	62.30 ± 6.46	66.70 ± 8.90	65.85 ± 9.47	0.113	0.65
Gender (*n*, %)				0.259	
Male	14 (51.9)	10 (32.3)	10 (50.0)		-
Female	13 (48.1)	21 (67.7)	10 (50.0)		-
Education (years)	11.74 ± 2.90	11.55 ± 3.00	9.55 ± 3.79^a,b^	0.046	0.68
Hypertension	12 (44.4)	12 (38.7)	5 (25.0)	0.385	-
Hyperlipidemia	3 (11.1)	7 (22.6)	1 (5.0)	0.182	-
Diabetes	4 (14.8)	6 (19.4)	0 (0.0)	0.121	-
MMSE (M,IQR)	29 (2)	27 (1)	19.5 (11.25)^a,b^	<0.001	-
MOCA (M,IQR)	26 (3)	22 (5)^a^	14 (6.75)^a,b^	<0.001	-
HAMD	5.67 ± 5.13	5.03 ± 3.97	4.50 ± 3.58	0.653	0.95
ADL (M, IQR)	8 (0)	8 (0)	10.5 (5.75)^a,b^	<0.001	-
CDR (M, IQR)	0 (0)	0.5 (0)^a^	1 (1)^a,b^	<0.001	-
CDR = 0	27 (100)	0	0		-
CDR = 0.5	0	31 (100)	1 (5)		-
CDR = 1	0	0	10 (50)		-
CDR = 2	0	0	5 (25)		–
CDR = 3	0	0	4 (20)		-
AVLT-DR	6.32 ± 1.89	2.23 ± 1.65^a^	0.65 ± 1.14^a,b^	<0.001	0.97
AVLT-R	21.33 ± 1.96	17.52 ± 2.56^a^	11.05 ± 5.78^a,b^	<0.001	0.99
Z-Lang	0.45 ± 0.46	0.16± 0.76^a^	−0.85 ± 0.67^a,b^	<0.001	0.997
Z-Executive	0.7^η^ ± 0.85	0.6^η^ ± 0.76	/	0.271	-
Z-Processing speed	0.4^η^ ± 0.87	0.3^η^ ± 0.82	/	0.846	-

*Data are represented as mean ± SD or median (IQR) or n (%); a: vs. NC P < 0.05; b: vs. aMCI P < 0.05; η, data × 10^−4^ equal to original data; Z-Lang, Z standardized language ability; Z-EF, Z standardized executive function; Z-proc, Z standardized processing speed; MMSE, mini-mental state examination; MoCA, Montreal cognitive assessment; CDR, clinical dementia rating; AVLT-DR, auditory verbal learning test delayed recall; AVLT-R, auditory verbal learning test recognition; HAMD, Hamilton depression rating scale; ADL, activities of daily living scale. aMCI, amnestic mild cognitive; AD, Alzheimer's disease; NC, normal control*.

### TBSS Analyses Between Groups and *post-hoc* Analysis

**Figure 1:**
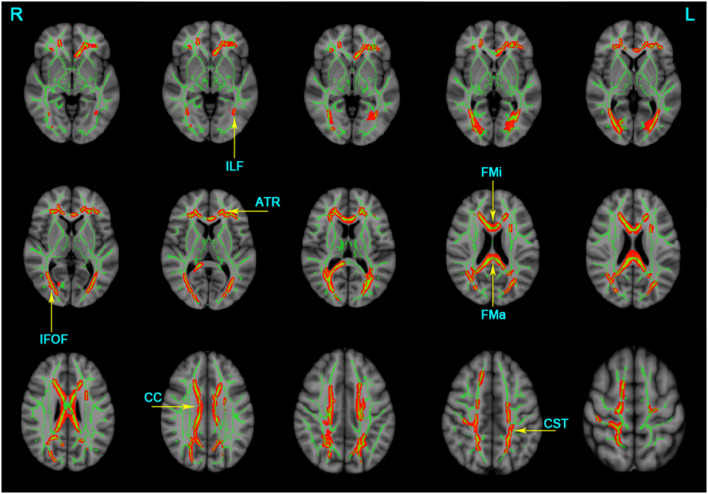
Clusters with decreased FA in AD patients by TBSS analysis. Significant clusters (red, threshold-free cluster enhancement corrected and Bonferroni corrected) in axial views overlaid onto the group averaged FA skeleton (green) and the MNI152 T1 template; ATR, anterior thalamic radiation; CST, corticospinal tract; FMa, forceps major; FMi, forceps minor; IFOF, inferior fronto-occipital fasciculus; ILF, inferior longitudinal fasciculus; CC, corpus callosum; L, left; R, right. FA, fractional anisotropy.

The TBSS analyses revealed widespread WM microstructure changes among the three groups. For reduced FA, the anatomical locations and clusters voxel were listed in Table 2, including the bilateral ATR, CST, IFOF, ILF, and unilateral for ma, for mi, right SLF, left UF, and CC. For each subject, the mean FA values across all voxels in each significant cluster were computed for further *post-hoc* analysis. Compared to the NC and aMCI groups, the AD group presented decreased FA values in the bilateral ATR, IFOF, ILF, and unilateral left CST, for ma, for mi, and CC (Bonferroni correction, *P* < 0.05) (Figure 1). No significant differences in FA values were found between the NC and aMCI groups.

**Table 2:** Significant clusters of FA values among the three groups by TBSS analysis.

**Anatomical extent of cluster^#^^,^^&^**	**Cluster voxel**	**NC**	**aMCI**	**AD**	***P***	***Power***
ATR-L	100	0.342 ± 0.038	0.332 ± 0.056	0.289 ± 0.031^a,b^	<0.001^***^	0.994
ATR-R	128	0.348 ± 0.041	0.338 ± 0.043	0.298 ± 0.028^a,b^	<0.001^***^	0.972
CST-L	67	0.475 ± 0.034	0.472 ± 0.041	0.433 ± 0.041^a,b^	0.001^***^	0.986
CST-R	161	0.482 ± 0.037	0.5 ± 0.048	0.455 ± 0.041^b^	0.002^***^	0.967
For ma	598	0.668 ± 0.02	0.661 ± 0.036	0.611 ± 0.025^a,b^	<0.001^***^	1.0
For mi	1099	0.55 ± 0.031	0.541 ± 0.036	0.5 ± 0.026^a,b^	<0.001^***^	1.0
IFOF-L	295	0.49 ± 0.023	0.486 ± 0.044	0.438 ± 0.022^a,b^	<0.001^***^	1.0
IFOF-R	285	0.51 ± 0.03	0.505 ± 0.044	0.454 ± 0.035^a,b^	<0.001^***^	1.0
ILF-L	88	0.539 ± 0.034	0.548 ± 0.066	0.481 ± 0.035^a,b^	<0.001^***^	1.0
ILF-R	58	0.53 ± 0.037	0.528 ± 0.045	0.479 ± 0.044^a,b^	<0.001^***^	0.997
SLF-R	20	0.481 ± 0.038	0.481 ± 0.06	0.448 ± 0.06	0.062	0.845
UF-L	18	0.375 ± 0.035	0.373 ± 0.037	0.346 ± 0.029	0.01	0.778
CC	3401	0.642 ± 0.029	0.629 ± 0.047	0.582 ± 0.039^a,b^	<0.001^***^	1.0

& *Significant clusters from TBSS results among three groups through a GLM (corrected by threshold-free cluster enhancement, P < 0.01); FA values are presented as means ± SD*;

*** *P < 0.05 after Bonferroni correction. a: vs. NC P < 0.05 (Bonferroni correction); b: vs. aMCI P < 0.05 (Bonferroni correction)*;

# *Anatomical locations were defined from JHU ICBM-DTI-81 white-matter labels and JHU WM tractography atlas. ATR, anterior thalamic radiation; CST, corticospinal tract; For ma, forceps major; For mi, forceps minor; IFOF, inferior fronto-occipital fasciculus; ILF, inferior longitudinal fasciculus; SIF, superior longitudinal fasciculus; UF, uncinate fasciculus; CC, corpus callosum; L, left; R, right; aMCI, amnestic mild cognitive; AD, Alzheimer's disease. NC, normal control; FA, fractional anisotropy*.

**Figure 2:**
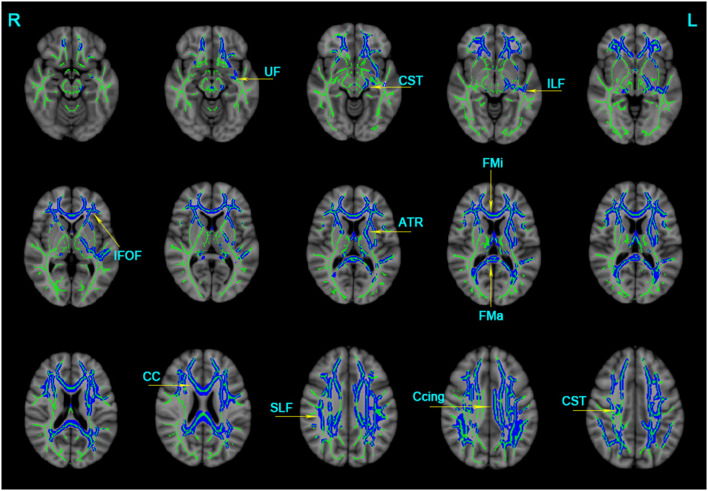
Clusters with increased MD in AD patients by TBSS analysis. Significant clusters (blue, threshold-free cluster enhancement corrected and Bonferroni corrected) in axial views overlaid onto the averaged MD skeleton of all participants (green) and the MNI152 T1 template; ATR, anterior thalamic radiation; CST: corticospinal tract; Ccing: cingulum (cingulate gyrus); FMa: forceps major; FMi: forceps minor; IFOF, inferior fronto-occipital fasciculus; ILF, inferior longitudinal fasciculus; SIF, superior longitudinal fasciculus; UF, uncinate fasciculus; CC, corpus callosum; L: left; R: right; MD, mean diffusivity.

The significant clusters of MD values among the three groups were listed as follows: the bilateral ATR, CST, Ccing, IFOF, SLF, UF, and unilateral for ma, for mi, CC, and left ILF (Table 3). In contrast to the NC group, the AD group displayed increased MD in the bilateral ATR, CST, IFOF, SLF, UF, and unilateral for ma, for mi, CC, left ILF, and left Ccing (Bonferroni correction, *P* < 0.05) (Figure 2). No significant changes in MD values were found in aMCI and NC.

**Table 3:** Significant clusters of MD values among the three groups by TBSS analysis.

**Cluster^#^^,^^&^**	**Cluster voxel**	**NC**	**aMCI**	**AD**	***P***	***Power***
ATR-L	628	0.819 ± 0.05	0.848 ± 0.085	0.964 ± 0.077^a,b^	<0.001^***^	1.0
ATR-R	353	0.811 ± 0.058	0.823 ± 0.084	0.95 ± 0.067^a,b^	<0.001^***^	1.0
CST-L	568	0.708 ± 0.02	0.717 ± 0.019	0.753 ± 0.03^a,b^	<0.001^***^	1.0
CST-R	126	0.725 ± 0.019	0.736 ± 0.03	0.762 ± 0.038^a,b^	<0.001^***^	0.99
Ccing-L	219	0.726 ± 0.023	0.729 ± 0.04	0.781 ± 0.039^a,b^	<0.001^***^	1.0
Ccing-R	5	0.703 ± 0.032	0.712 ± 0.051	0.741 ± 0.048	0.014	0.96
For ma	333	0.772 ± 0.029	0.784 ± 0.039	0.83 ± 0.03^a,b^	<0.001^***^	0.99
For mi	3358	0.75 ± 0.031	0.772 ± 0.048	0.817 ± 0.04^a,b^	<0.001^***^	0.99
IFOF-L	691	0.745 ± 0.026	0.754 ± 0.041	0.8 ± 0.039^a,b^	<0.001^***^	0.99
IFOF-R	462	0.731 ± 0.029	0.738 ± 0.039	0.784 ± 0.035^a,b^	<0.001^***^	1.0
ILF-L	87	0.818 ± 0.035	0.811 ± 0.035	0.859 ± 0.04^a,b^	<0.001^***^	0.995
SLF-L	1278	0.733 ± 0.026	0.732 ± 0.031	0.774 ± 0.027^a,b^	<0.001^***^	1.0
SLF-L	428	0.729 ± 0.02	0.729 ± 0.032	0.764 ± 0.03^a,b^	<0.001^***^	1.0
UF-L	142	0.737 ± 0.02	0.756 ± 0.032	0.784 ± 0.033^a,b^	<0.001^***^	1.0
UF-R	8	0.714 ± 0.026	0.719 ± 0.04	0.75 ± 0.03^a,b^	<0.001^***^	0.995
CC	5093	0.832 ± 0.03	0.859 ± 0.062	0.918 ± 0.046^a,b^	0.001^***^	1.0

& *Significant clusters from TBSS results among three groups through a GLM (corrected by threshold-free cluster enhancement, P < 0.01); MD data reflect the original values × 10^−3^ and are presented as means ± SD*;

*** *P < 0.05 after Bonferroni correction. a: vs. NC P < 0.05 (Bonferroni correction); b: vs. aMCI P < 0.05 (after Bonferroni correction)*;

# *Anatomical locations were defined from JHU ICBM-DTI-81 white-matter labels and JHU WM tractography atlas. ATR, anterior thalamic radiation; CST, corticospinal tract; Ccing, cingulum (cingulate gyrus); For ma, forceps major; For mi, forceps minor; IFOF, inferior fronto-occipital fasciculus; ILF, inferior longitudinal fasciculus; SIF, superior longitudinal fasciculus; UF, uncinate fasciculus; CC: corpus callosum; L, left; R, right; aMCI, amnestic mild cognitive; AD, Alzheimer's disease. NC, normal control, MD, mean diffusivity*.

**Figure 3:**
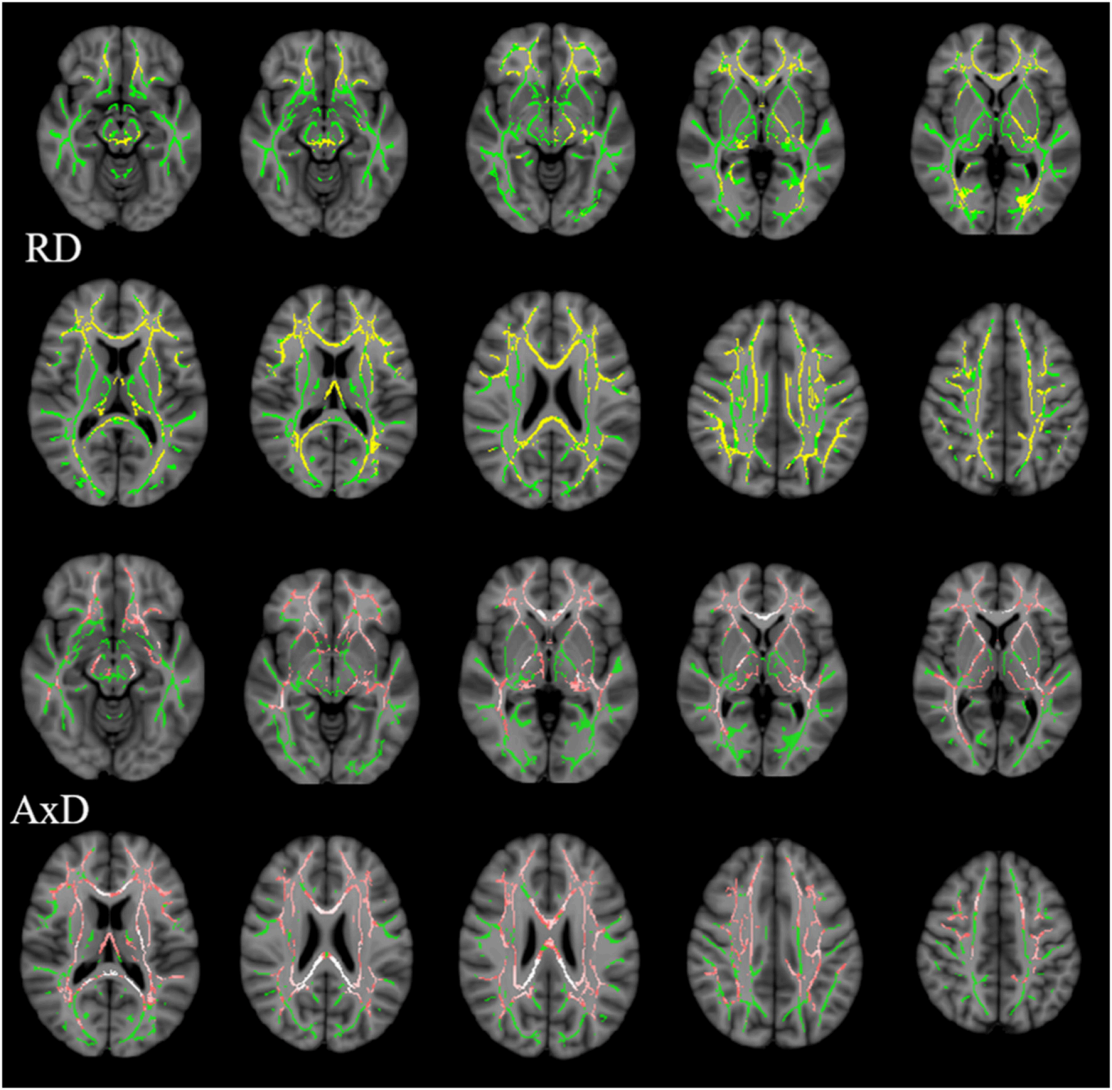
Clusters with increased RD/AxD in AD patients in TBSS analysis. Significant clusters (significant RD clusters are in yellow, significant AxD clusters are in pink, threshold-free cluster enhancement corrected and Bonferroni corrected) in axial views overlaid onto the averaged FA skeleton of all participants (green) and the MNI152 T1 template; RD, radial diffusion; AxD, axial diffusion.

Multiple tracts were identified where elevated RD reached significant levels in AD patients, including the bilateral ATR, CST, IFOF, ILF, SLF, and for ma, for mi, and left Ccing (Bonferroni correction, *P* < 0.05). Compared with the NC group, significant elevated RD values were found (Table 4, Figure 3) in the tract of the right CST, right IFOF, right ILF, and for mi in the aMCI group. Likewise, significant elevated AxD values were identified (Table 5, Figure 3) in the bilateral ATR, ILF, SLF, and left CST, left IFOF, right UF, for ma, and for mi. As for AxD value, there was no difference between the aMCI and NC groups.

**Table 4:** Significant clusters of RD values among three groups by TBSS analysis.

**Cluster^#^^,^^&^**	**Cluster voxel**	**NC**	**aMCI**	**AD**	***P***	***Power***
ATR-L	523	0.649 ± 0.053	0.68 ± 0.093	0.802 ± 0.077^a,b^	<0.001^***^	1.0
ATR-R	288	0.646 ± 0.066	0.668 ± 0.093	0.807 ± 0.068^a,b^	<0.001^***^	1.0
CST-L	646	0.467 ± 0.025	0.476 ± 0.025^a^	0.514 ± 0.035^a,b^	<0.001^***^	1.0
CST-R	311	0.519 ± 0.027	0.525 ± 0.035	0.56 ± 0.036^a,b^	<0.001^***^	0.999
Ccing-L	226	0.446 ± 0.034	0.452 ± 0.044	0.514 ± 0.054^a,b^	<0.001^***^	1.0
Ccing-R	21	0.5 ± 0.039	0.518 ± 0.043	0.548 ± 0.064^a,b^	0.005	0.965
For ma	621	0.413 ± 0.025	0.424 ± 0.045	0.481 ± 0.038^a,b^	<0.001^***^	1.0
For mi	3254	0.512 ± 0.032	0.531 ± 0.049^a^	0.579 ± 0.041^a,b^	<0.001^***^	1.0
IFOF-L	705	0.545 ± 0.027	0.55 ± 0.047	0.604 ± 0.039^a,b^	<0.001^***^	1.0
IFOF-R	664	0.544 ± 0.028	0.551 ± 0.044^a^	0.602 ± 0.035^a,b^	<0.001^***^	1.0
ILF-L	126	0.533 ± 0.03	0.527 ± 0.058	0.588 ± 0.05^a,b^	<0.001^***^	1.0
ILF-R	44	0.511 ± 0.03	0.511 ± 0.042^a^	0.552 ± 0.045^a,b^	0.001^***^	0.998
SLF-L	653	0.547 ± 0.031	0.538 ± 0.031	0.58 ± 0.033^a,b^	<0.001^***^	0.993
SLF-L	187	0.531 ± 0.031	0.53 ± 0.04	0.572 ± 0.039^a,b^	<0.001^***^	0.997

& *Significant clusters from TBSS results among three groups through a GLM (corrected by threshold-free cluster enhancement, P < 0.01); RD data equal to the original values × 10^3^ and are presented as means ± SD*;

*** *P < 0.05 after Bonferroni correction. a: vs. NC P < 0.05; b: vs. aMCI P < 0.05*;

# *Anatomical locations were defined from the JHU WM tractography atlas. ATR, anterior thalamic radiation; CST, corticospinal tract; Ccing, cingulum (cingulate gyrus); For ma, forceps major; For mi, forceps minor; IFOF, inferior fronto-occipital fasciculus; ILF, inferior longitudinal fasciculus; SIF, superior longitudinal fasciculus; L, left; R, right; aMCI, amnestic mild cognitive; AD, Alzheimer's disease. NC, normal control, RD, radial diffusion*.

**Table 5:** Significant clusters of AxD values among three groups by TBSS analysis.

**Anatomical extent of cluster^#^^,^^&^**	**Cluster voxel**	**NC**	**aMCI**	**AD**	***P***	***Power***
ATR-L	748	1.21 ± 0.058	1.236 ± 0.092	1.361 ± 0.075^a,b^	<0.001^***^	1.0
ATR-R	498	1.202 ± 0.068	1.21 ± 0.101	1.346 ± 0.11^a,b^	<0.001^***^	1.0
CST-L	398	1.199 ± 0.035	1.229 ± 0.058	1.287 ± 0.061^a,b^	<0.001^***^	1.0
CST-R	362	1.185 ± 0.032	1.208 ± 0.06	1.213 ± 0.034^a,b^	0.084	0.84
For ma	197	1.666 ± 0.066	1.686 ± 0.08	1.747 ± 0.073^a,b^	0.001^***^	0.971
For mi	2430	1.243 ± 0.042	1.276 ± 0.057	1.325 ± 0.042^a,b^	<0.001^***^	1.0
IFOF-L	646	1.141 ± 0.028	1.151 ± 0.048	1.206 ± 0.037^a,b^	<0.001^***^	1.0
IFOF-R	781	1.238 ± 0.034	1.21 ± 0.056	1.207 ± 0.054^a,b^	0.053	0.898
ILF-L	60	1.327 ± 0.046	1.309 ± 0.058	1.375 ± 0.056^a,b^	<0.001^***^	0.996
ILF-R	64	1.384 ± 0.055	1.329 ± 0.091	1.296 ± 0.084^a,b^	0.001^***^	0.999
SLF-R	1080	1.116 ± 0.037	1.138 ± 0.048	1.212 ± 0.037^a,b^	<0.001^***^	1.0
SLF-L	551	1.122 ± 0.032	1.139 ± 0.054	1.195 ± 0.04^a,b^	<0.001^***^	1.0
UF-L	172	1.2 ± 0.038	1.227 ± 0.042	1.27 ± 0.052^a,b^	<0.001^***^	1.0

& *Significant clusters from TBSS results among three groups through a GLM (corrected by threshold-free cluster enhancement, P < 0.01); AxD data equal to the original values × 10^3^ and are presented as means ± SD*;

*** *P < 0.05 after Bonferroni correction. a: vs. NC P < 0.05; b: vs. aMCI P < 0.05*;

# *Anatomical locations were defined from the JHU WM tractography atlas. ATR, anterior thalamic radiation; CST, corticospinal tract; Ccing, cingulum (cingulate gyrus); For ma, forceps major; For mi, forceps minor; IFOF, inferior fronto-occipital fasciculus; ILF, inferior longitudinal fasciculus; SIF, superior longitudinal fasciculus; L, left; R, right; aMCI, amnestic mild cognitive; AD, Alzheimer's disease. NC, normal control, AxD, axial diffusion*.

### Correlation Between Cognitive Domains and Diffusion Metrics in the aMCI or AD Group

**Figure 4:**
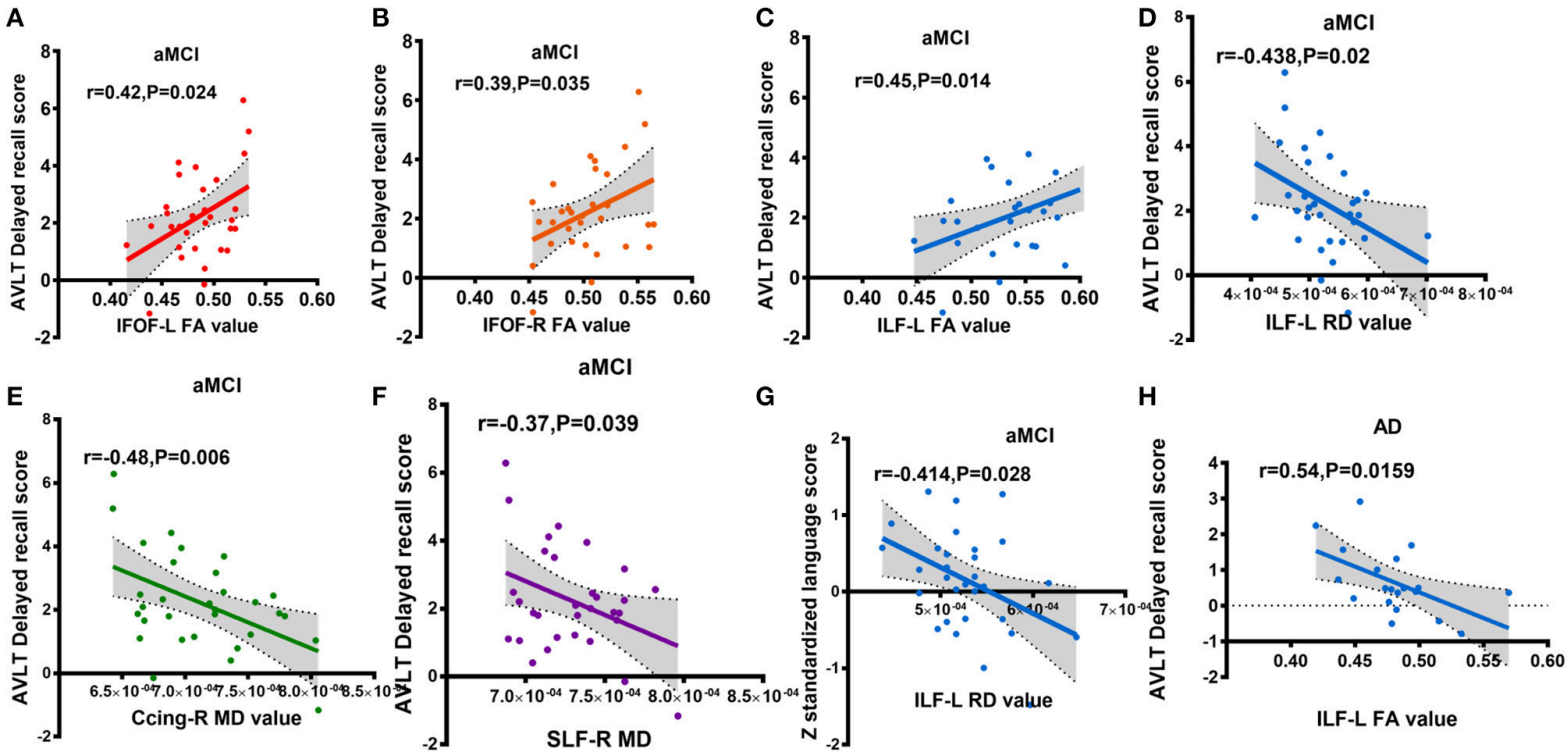
Partial correlations between cognitive scores and altered diffusion value, adjusted for years of education, age, and gender (A-G: aMCI group; H: AD group); **(A)** correlation between IFOF-L FA value and AVLT delayed recall score; **(B)** correlation between IFOF-R FA value and AVLT delayed recall score; **(C)** correlation between ILF-L FA value and AVLT delayed recall score; **(D)** correlation between ILF-L RD value and AVLT delayed recall score; **(E)** correlation between Ccing-R MD value and AVLT delayed recall score; **(F)** correlation between SLF-R MD value and AVLT delayed recall score; **(G)** correlation between ILF-L RD value and z standardized language AVLT delayed recall score; **(H)** correlation between ILF-L FA value and AVLT delayed recall score. Ccing, cingulum (cingulategyrus); IFOF, inferior fronto-occipital fasciculus; ILF, inferior longitudinal fasciculus; SIF, superior longitudinal fasciculus; L, left; R, right. FA, fractional anisotropy; MD, mean diffusivity; RD, radial diffusion aMCI, amnestic mild cognitive; AD, Alzheimer's disease; AVLT-DR, auditory verbal learning test delayed recall. Different colors represent different fibers.

For the aMCI group, significant correlations were found between the mean FA value of ROI and AVLT-DR scores in the following tracts: left IFOF (*r* = 0.42, *P* = 0.024, Figure 4A), right IFOF (*r* = 0.39, *P* = 0.035, Figure 4B), and left ILF (*r* = 0.45, *P* = 0.014, Figure 4C). The mean MD value of ROI in the right Ccing (*r* = −0.48, *P* = 0.006, Figure 4E) and right SLF (*r* = −0.37, *P* = 0.039, Figure 4F) was negatively associated with the AVLT-DR scores. The average RD value of ROI in the left ILF was negatively correlated with the AVLT-DR scores (*r* = −0.438, *P* = 0.02, Figure 4D) and language function (*r* = −0.414, *P* = 0.028 Figure 4G). Moreover, the left ILF FA value had a negative correlation with the AVLT-DR scores in the AD group (*r* = −0.54, *P* = 0.0159, Figure 4H). It is worth pointing out that the correlations all listed above were not able to survive Bonferroni correction. No significant correlations were observed between other cognitive domains and DTI metrics in both the aMCI and AD groups.

### DTI Metrics Separate AD Patients From Other Participants by SVM Classification

**Figure 5:**
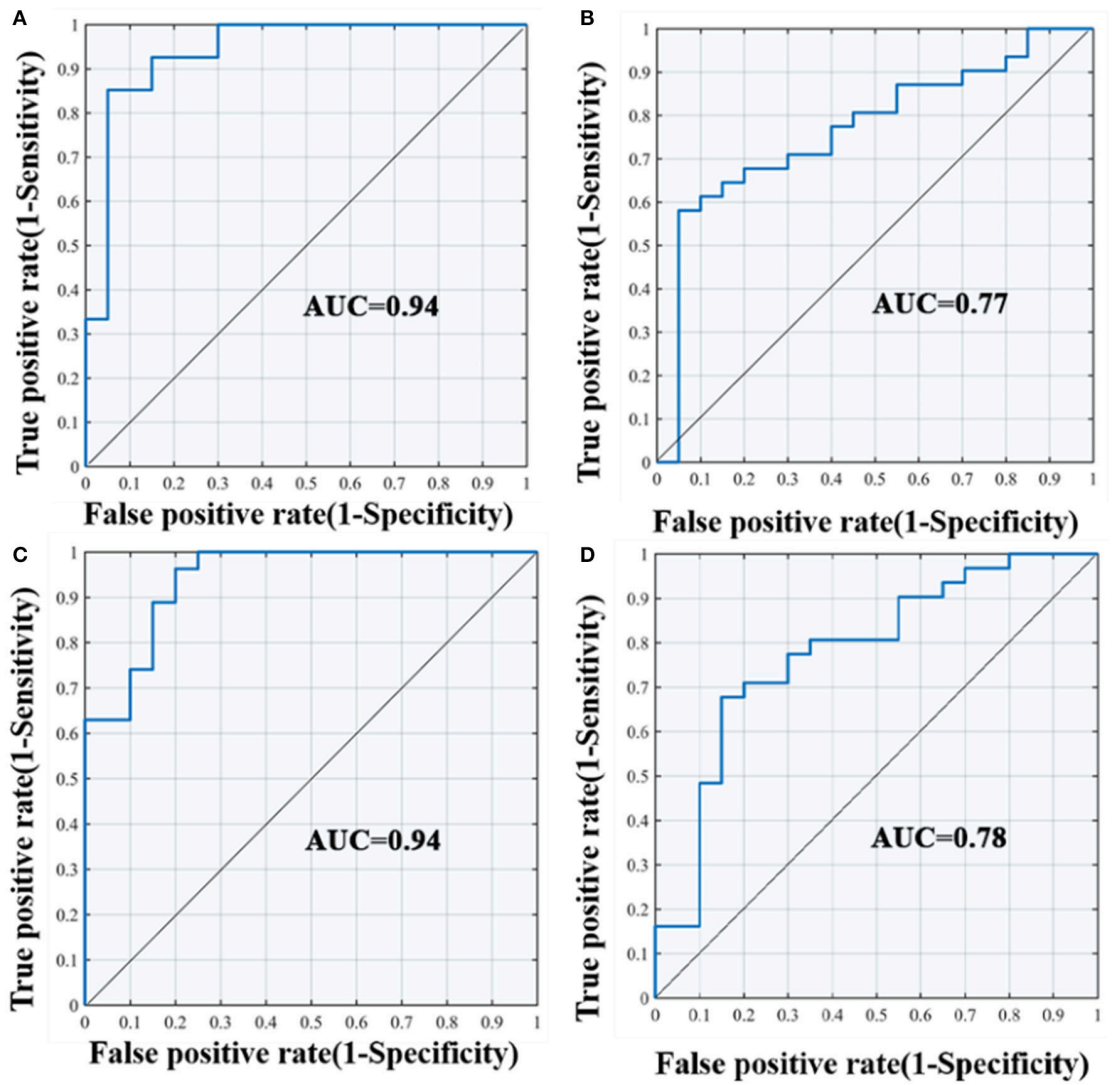
ROC curves of the SVM classification between groups. **(A)** differentiate the AD and NC group according to FA value; **(B)** differentiate the AD and aMCI group according to FA value; **(C)** differentiate the AD and NC group according to MD value; **(D)** differentiate the AD and aMCI group according to MD value; AUC, area under curve; SVM, support vector machine; ROC, receiver operating characteristic curve.

The results of the SVM classification demonstrated the discriminative power of disrupted diffusion metrics in the differentiation of AD patients from the other two groups (Figure 5). The disrupted FA value exhibited an area under curve (AUC) of 0.94 (accuracy 89%, sensitivity 85%, specificity 93%, Figure 5A) for discriminating AD patients from controls, and an AUC of 0.77 (accuracy 69%, sensitivity 70%, specificity 68%, Figure 5B) for discrimination between AD and aMCI. The disrupted MD value showed an AUC of 0.94 (accuracy 85%, sensitivity 85%, specificity 85%, Figure 5C) for discrimination between AD and controls, and an AUC value of 0.78 (accuracy 73%, sensitivity 65%, specificity 78%, Figure 5D) for discrimination between AD and aMCI. It should be noted that the identifying accuracy of aMCI from control participants was lower than 50% for both the FA and MD value.

## Discussion

This current study investigated the potential roles of DTI metrics in the discriminative ability of AD-spectrum patients. Using TBSS and SVM classification approaches, three observations were made: (i) limbic association tracts, cortico-cortical, interhemispheric, and corticospinal tracts were widely damaged in AD patients; (ii) the long longitudinal tracts (including IFOF, ILF, and SLF in our study) are closely related to memory function in aMCI or AD patients. (iii) DTI metrics of the WM microstructure allowed for the classification of these samples, indicating their potential role as “trait” neuroimaging markers for monitoring the progression of AD.

Relative to normal elderly adults, AD patients displayed decreased FA values and increased MD/RD/AxD values in widespread brain regions, including temporoparietal regions and the fronto-parietal cortex. The aMCI group showed a tendency topographic concordant with the AD group. Previous DTI studies have demonstrated that WM alterations in clinical AD spectrum disease were initially localized in the medial temporal limbic associated tracts, then prominently spread to the temporal and parietal, and to a lesser extent, the frontal lobes which was associated with WM as the disease progressed (31–33). The pattern of WM change is consistent with the pathology of AD which primarily involves the temporal lobe. In the present study, the WM integrity of AD patients decreased in the bilaterally limbic tracts (UF and Ccing), which were in line with previous literatures (34, 35). The UF is a WM tract connecting the anterior part of the temporal lobe with the frontal lobe which is associated with episodic memory. A 3-years follow-up research considered WM abnormality in UF as a potential indicator of the conversion from aMCI to AD (36). The cingulum occupying the central position of the papez circuit is assumed to be vital for normal memory functions (37). UF and cingulum tract damage would lead to memory decline. In addition, impaired CC was also observed in the dementia group. CC is the main connectivity pathway between hemispheres. Any neurodegenerative process involving the cerebral WM may have an impact on the diffusivity of CC (31). Our findings of destruction in the IFOF, ILF, and SLF in the aMCI and AD groups were consistent with previous ROI-based and voxel-based DTI studies, which showed damage of the WM regions outside the medial temporal lobe (MTL) network in AD patients (34). Our results supported the hypothesis that AD had a disconnection process (38).

There are many theories about the underlying pathophysiology of WM damage (39). Previous studies observed Wallerian degeneration secondary to neuronal damage in the AD brains postmortem (40). Pathological vascular deposition of amyloid protein β (Aβ) might play a role in the microvascular alterations and WM lesions in AD through neuroinflammation (39–41). A large body of literature on prior DTI work has shown neurodegenerative changes of brain WM in aMCI when compared to healthy controls (42–44), typically in the posterior cingulate gyrus and hippocampus (45, 46). In the present study, we failed to observe cingulate gyrus and hippocampus diffusion metric differences in the aMCI subjects. Nonetheless, the mean RD value in ROI of the for mi, right IFOF, and left ILF have shown significant differences in the aMCI group when compared with normal control. It might be due to the fact that the patients in our cohort are in the extremely early stage of aMCI, and the cognitive impairment was relatively slight.

When considering the relation between cognitive domains and DTI metrics, the scores of memory performance revealed significant correlations with the FA, MD, and RD index in certain specific fiber bundles in the present aMCI or AD group. Recent research has shown that associations between the WM microstructure and memory or executive function were evident across widespread regions of the brain in a mixed sample of AD and healthy elderly subjects (47). However, no significant linear relationship between the degree of WM disruption and the level of cognitive function (memory and executive abilities) were found within either the AD or healthy older adult group (47). In our study within the aMCI group, AVLT-DR scores were positively related to the mean FA value of significant clusters in the bilateral IFOF and left ILF; and negatively related to the mean MD value of significant clusters in the right Ccing and right SLF. The IFOF served as a direct connection from the extra-striata occipital cortex to the anterior temporal lobe, playing an important role in visuospatial processing, object recognition, and memory (46). Previous tractography studies in aMCI and AD have shown diffusion abnormalities in the IFOF (34, 48). We further confirmed its close relationship to memory function in the preclinical phase of dementia. Meanwhile, the relationship between the IFOF, the cingulum tracts, and memory performance indicated that memory function depends on both the integrity of MTL structures and their connectivity with temporal, parietal, and frontal lobe regions. On the contrary, the FA value in the left ILF was negatively related to the AVLT-DR scores in the AD group. Namely, some subjects with higher FA values suffered from poorer memory function. It could be the results of dispersed WM impairment and gray matter atrophy in the AD group.

MTL substructures are the earliest regions affected by AD pathology, mainly amyloid deposition, and neurofibrillary tangle tau pathology. Anatomically, WM degeneration in AD follows the topographic progression of cortical AD pathology (49, 50). As mentioned above, AD pathological invasion was initially localized in MTL, and then gradually spread to the temporal, parietal, and frontal lobes. In the current study, the early stage of the aMCI group showed WM disruption in the right IFOF and left ILF as reflected by the RD property. While in the dementia stage, more extensive and severe white matter damage was observed. IFOF has complex intrinsic projections of the brain, including extensive cortical termination territories within the middle and superior frontal, middle and posterior temporal lobes, superior parietal, and angular gyri (51, 52). This means that the diffusion abnormalities of IFOF could influence the structural connectivity architecture among the MTL, the frontal lobe, and the parietal lobe (53). Besides, the ILF also has projections involving the MTL (54). Therefore, we speculated that in the early stages, long fibers pathway damage could indirectly affect the MTL connectivity, which can be used to explain early memory loss. The mediation role of long longitudinal tract alterations during the progression of AD needs to be investigated in more large-scale cohorts.

SVM provides great potential for future artificial intelligence aided diagnosis, and has been increasingly implemented in various classification problems (49). For instance, SVM successfully classified multiple sclerosis patients from healthy controls with accuracies as high as 89% by combined DTI and functional data (50). Similarly, we revealed that combining disrupted diffusion metrics and SVM classification, the accuracy of classification was higher than 85% in distinguishing AD patients from controls, which shows promise comparable with previous brain microstructure studies to predict progression (55, 56). A moderate discernment was observed between the AD patients and aMCI with the accuracy of 69 and 73%. We did fail to distinguish with high accuracy (lower than 50%) between aMCI patients with the NC. This could be due to the little difference between cognitive levels between both groups. It may also be due to insufficient sample size which affects the classification efficiency. These findings shed light on the potential utility of brain WM microstructural-based markers combined with SVM classification for the individual prediction of disease progression, which may provide a novel avenue into the early diagnosis of AD.

Several limitations of the study should be considered. Firstly, our small sample may limit the statistical power of the differences of WM integrity between groups. Replication in an independent and larger cohort is necessary. Secondly, our diagnosis of aMCI was purely based on clinical symptoms. The sample would be less heterogeneous if biomarkers were added into the inclusion criteria. Thirdly, our research mainly focused on WM microstructure alteration. Future research should investigate the association between WM alterations, gray matter atrophy, metabolic function changes, and pathological indicators to offer a comprehensive insight into the pathologic basis of WM neurodegeneration.

The original intention of this study was to analyze the integrity of the whole brain WM in patients on the AD spectrum and hoped that the machine learning classification could suitably classify the disease according to the characteristics of WM damage. We initially explored the role of mechanical classification in assisting clinical diagnosis of AD diseases to make up for the drawbacks of subjectivity in assessment by the artificial cognition scale and to compensate for the shortcomings of being easily affected by education level and cultural background. This study found that consistent with previous reports, AD patients have extensive WM damage. Long connective fibers lesions are obviously associated with memory deficiency. The effect of mechanical classification in the early stage of the disease is not very satisfactory, but it is very favorable in the AD stage.

## Conclusion

The WM microstructure is extensively disrupted in AD. An impaired long longitudinal tract (including IFOF, ILF, and SLF) is closely related to memory deficits. DTI metrics of the brain WM microstructure may hold potential value for the diagnosis of AD as an imaging biomarker. The classification effect of SVM in the early stages of AD disease needs to be further exploration.

## Data Availability Statement

The datasets generated for this study are available on request to the corresponding author.

## Ethics Statement

The research protocol received approval from ethics committee of the Affiliated Drum Tower Hospital of Nanjing University Medical School (clinical trials government identifier: NCT01364246) and each participant provided written informed consent prior to any experimental procedures.

## Author Contributions

HZ and FB designed the study, collected the data, and edited the manuscript. YX designed the study and edited the manuscript. CL collected the data, wrote and edited the manuscript, and performed the statistics. RQ wrote and edited the manuscript. ML, DY, LH, and RL collected the data. HC and QY validated the statistics. All authors contributed to the article and approved the submitted version.

## Conflict of Interest

The authors declare that the research was conducted in the absence of any commercial or financial relationships that could be construed as a potential conflict of interest.
